# Aurones: A Promising Heterocyclic Scaffold for the Development of Potent Antileishmanial Agents

**DOI:** 10.1155/2012/196921

**Published:** 2012-09-25

**Authors:** Marina Roussaki, Sofia Costa Lima, Anna-Maria Kypreou, Panagiotis Kefalas, Anabela Cordeiro da Silva, Anastasia Detsi

**Affiliations:** ^1^Laboratory of Organic Chemistry, School of Chemical Engineering, National Technical University of Athens, Heroon Polytechniou 9, Zografou Campus, 15780 Athens, Greece; ^2^Parasite Disease Group, IBMC-Institute for Molecular and Cell Biology, University of Porto, Rua do Campo Alegre, 823, 4150-180 Porto, Portugal; ^3^Department of Food Quality and Chemistry of Natural Products, Mediterranean Agronomic Institute of Chania, International Centre for Advanced Mediterranean Agronomic Studies, 73100 Chania, Crete, Greece; ^4^Department of Biological Sciences, Faculty of Pharmacy, University of Porto, Rua de Jorge Viterbo Ferreiera, 228, 4050-313 Porto, Portugal

## Abstract

A series of (*Z*)-2-benzylidenebenzofuran-3-(2H)-ones (aurones) bearing a variety of substituents on rings A and B were synthesized and evaluated for their antiparasitic activity against the intracellular amastigote form of *Leishmania infantum* and their cytotoxicity against human THP1-differentiated macrophages. In general, aurones bearing no substituents on ring A (compounds **4a–4f**) exhibit higher toxicity than aurones with 4,6-dimethoxy substitution (compounds **4g–4l**). Among the latter, two aurones possessing a 2′-methoxy or a 2′-methyl group (compounds **4i** and **4j**) exhibit potent antileishmanial activity (IC_50_ = 1.3 ± 0.1 *μ*M and IC_50_ = 1.6 ± 0.2 *μ*M, resp.), comparable to the activity of the reference drug Amphotericin B, whereas they present significantly lower cytotoxicity than Amphotericin B as deduced by the higher selectivity index.

## 1. Introduction

Leishmaniasis, an infectious disease caused by protozoan parasites belonging to the genus *Leishmania* (*L*.), is transmitted to humans through the bite of female phlebotomine sand flies infected with the parasite.This disease is manifested in three forms: cutaneous leishmaniasis (CL), which is the most common form, mucocutaneous leishmaniasis (MCL), and visceral leishmaniasis (VL) [1]. CL causes ulcers on the exposed parts of the human body which leave permanent scars when healed, whereas MCL causes the destruction of the mucous membranes of the nose, mouth, and throat cavities and surrounding tissues leading to serious disabling of the patient. Although they do not cause death of the patient, these two forms pose a serious social problem as a result of prejudice or even rejection of the disfigured patient from the community. VL is the most serious form of the disease which inevitably leads to death if left untreated [2]. Leishmaniasis is distributed in four continents and affects millions of people, especially in the poor developing countries where the lack of efficient medical care and resources, malnutrition, and weak immune system enhance the possibility of infection [3]. Moreover, co-infection with HIV is an emerging threat as the immunosuppressed HIV patients are especially vulnerable to VL infection. In fact, the majority of the VL cases reported in southern Europe involves HIV-infected patients [4]. In addition, in developed countries, where leishmaniasis is not endemic, the cases of infected population have increased over the last decade, in association with increasing international tourism, military operations, and the influx of immigrants from endemic countries [5].

Treatment of leishmaniasis lies exclusively on chemotherapy because no human vaccine is yet available [6]. The first-line drugs are the pentavalent antimonials meglumine antimoniate (Glucantime) and sodium stibogluconate (Pentostam), which are now obsolete in India due to resistance development although they are still used in other countries [2, 7]. As second-line treatment, liposomal formulations of the polyene antibiotic Amphotericin B have been developed in order to increase efficacy and reduce toxicity of the drug [8]. However, although it has high cure rates, this drug suffers from severe side-effects and high cost which renders its use unaffordable for patients in developing countries. New, cheaper formulations of Amphotericin B are currently being explored as alternatives [9]. The first oral treatment for leishmaniasis has been developed in 2002 using miltefosine, an alkylphospholipid initially used as an anticancer agent [10]. Unfortunately, miltefosine has exhibited teratogenic potential; therefore its use is prohibited in pregnant women [11]. Current advances in leishmaniasis chemotherapy include paromomycin, an aminoglycoside antibiotic, which has recently started to be used as a treatment for CL and VL and sitamaquine, a 8-amino-quinolone that has reached phase II clinical trials for the treatment of VL [10, 12, 13].

The development of new, more effective, less toxic, and affordable antileishmanial drugs is still a major goal in medicinal chemistry based not only on rational drug design [14, 15], but also on the exploitation of nature either as a source of phytochemical antiparasitics or as an inspiration for new molecular scaffolds [16–19]. The evaluation of the antiparasitic activity of plant extracts or isolated natural products constitutes an attractive approach for the development of new efficient drugs.

**Figure 1:**
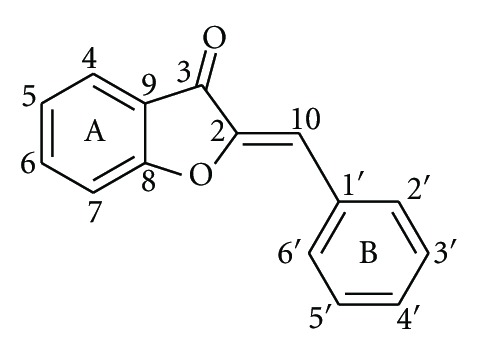
Chemical structure and numbering of the aurone scaffold.

Aurones, (*Z*)-2-benzylidenebenzofuran-3-(2H)-ones (Figure 1), constitute a subclass of flavonoids which occur rarely in nature. They are responsible for the bright yellow color of some popular ornamental flowers and are biosynthesized from chalcones by the key enzyme aureusidin synthase. Although the studies of the biological activities of aurones are still limited, these natural products and their synthetic analogues have proved to be promising bioactive compounds with a broad spectrum of activity including anticancer [20–22] antimicrobial, [23] and antioxidant [24, 25] properties whereas they possess enzyme inhibitory [25, 26], or enzyme-inducing activity [27].

The antileishmanial activity of a series of aurones was first reported by Kayser and Kiderlen in 1999 [28]. In this study, five aurones and one aurone glucoside were tested against extracellular promastigotes of *L. donovani*, *L. infantum*, *L. enriettii*, and *L. major*, and against intracellular amastigote* L. donovani,* and the results revealed that the compounds were active against the parasites in concentrations ranging between 0.4 and 5.0 *μ*g/mL while they showed moderate cytotoxicity. The same research group studied structurally diverse aurones and auronols for their inhibitory activity against the mitochondrial NADH-fumarate reductase of *L. major* promastigotes. The majority of the compounds were potent inhibitors of the enzyme in a dose- and structure-dependent manner [29].

As a continuation of our studies towards the synthesis and activity evaluation of novel bioactive natural product analogues, we have focused on the aurone scaffold in order to further explore its potential in the development of novel and efficient antileishmanial agents. In this work we describe the synthesis and structural characterization of aurone derivatives bearing various substituents on different positions of rings A and B as well as the evaluation of their activity against the intracellular form of *L. infantum* parasites.

## 2. Results and Discussion

### 2.1. Chemistry

**Scheme 1:**
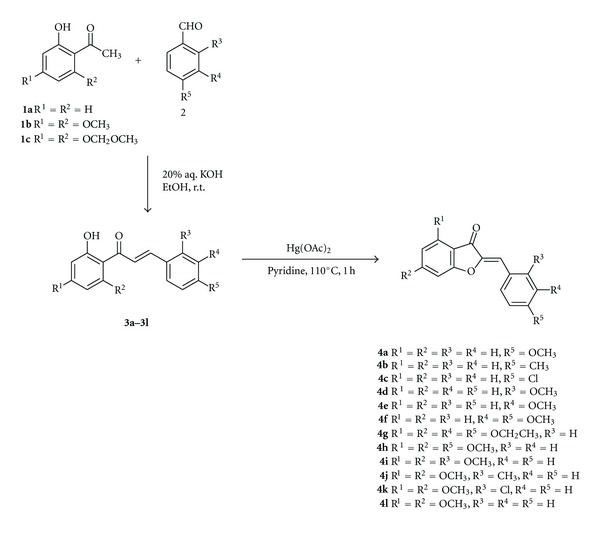
Synthesis of chalcones 3**a**–3**l**  and of the corresponding aurones 4**a**–4**l**.

Aurones **4a**–**4l** have been synthesized via the oxidative cyclization of the corresponding chalcones using mercury(II) acetate in pyridine. The synthesis and characterization of compounds **4a**–**4i **has been described in detail in one of our previous publications [25]. In order to accomplish the synthesis of aurones **4j**–**4l**, chalcones **3j**–**3l** were synthesized via the Claisen-Schmidt condensation reaction between 4′,6′-dimethoxy-2′-hydroxy-acetophenone (**1b**) and the appropriately substituted benzaldehyde in basic conditions (20% aqueous KOH in ethanol) (Scheme 1).

The oxidative cyclization of chalcones **3j**–**3l** proceeds smoothly by refluxing the corresponding chalcone with Hg(OAc)_2_ in pyridine for 1 h (Scheme 1). The desired aurones **4j**–**4l** were isolated as yellow solids after acidification with aqueous HCl 10%, extraction with an appropriate solvent, and purification.

The structural characterization of the synthesized compounds was achieved using ^1^H and ^13^C NMR spectroscopy as well as MS/ESI(+) spectroscopy. The ^1^H NMR spectra of chalcones **3j**–**3l** are characterized by a signal at a low field (~14 ppm) attributed to the proton of the 2′-OH group which is deshielded as a result of taking part in a strong intramolecular hydrogen bond with the neighbouring carbonyl group, and the pair of AB doublets owed to the vinylic protons, with *J*~15 Hz confirming the *E*-geometry of the double bond. In the case of chalcones **3j** and **3k**, the presence of a substituent on position 2 results in a significant downfield shift of the AB doublets (8.08 and 8.13 ppm for H_*β*_ and 7.81 and 7.87 ppm for H_*α*_, resp.) as compared to the corresponding chemical shifts in the ^1^H NMR spectrum of the naturally occurring chalcone flavokawain B **(3l)** (7.91 ppm for H_*β*_ and 7.79 ppm for H_*α*_). This difference should be attributed to the “*ortho*” effect, a phenomenon which renders the spectra of *ortho*-substituted aromatic compounds more complex than those of their *meta- *and *para*-substituted counterparts [30].

**Scheme 2:**
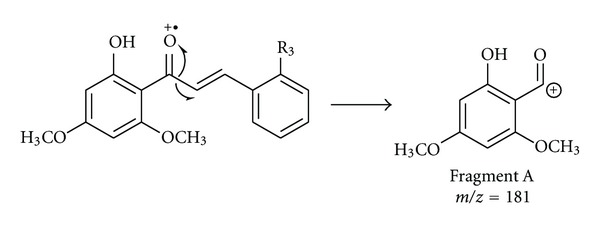
Major fragmentation pathway of chalcones 3**j**–3**l** in CID ESI-MS.

The mass spectra of chalcones **3j**–**3l **were obtained using the electrospray ionisation (ESI) technique and an ion trap detector. In the conditions of the ESI experiment, chalcones are protonated and the [M+H]^+^ ion is detected instead of the molecular ion. In the collision-induced dissociation (CID) mass spectra of the [M+H]^+^ cations of **3j**–**3l**, the fragmentation pattern observed involved a scission of the bond between the carbonyl carbon and the vinylic C_*α*_, which produced Fragment A as the basic ion (Scheme 2).

In general, the ^1^H NMR spectra of aurones are characterized by the presence of a singlet owed to the exocyclic vinylic proton, the chemical shift of which is indicative of the geometrical configuration of the molecule and is affected significantly by the position of the substituents on ring B and, to a lesser extent, by the presence of alkoxy substituents on ring A. In the case of the synthesized aurones **4a**–**4c** and **4e**–**4h**, **4l,** this singlet appears in the range of 6.57–6.89 ppm [25], in accordance with the previous findings by us and other research groups [21, 24, 25]. The chemical shift of the vinylic proton in the case of aurones **4d**, **4i**–**4k **which bear a substituent on position 2′ is observed downfield, at 7.00–7.49 ppm. Although some controversy was at first aroused as to if the downfield shift of vinylic H indicates the presence of the *E*-geometrical isomer of 2′-substituted aurones, the X-ray crystallographic analyses of two aurones reported recently proved that it is undoubtedly that the thermodynamically more stable *Z*-isomer always prevails [21, 31]. As a result, the difference in chemical shifts is attributed to the *ortho*-effect.

The observation of only one signal owed to the vinylic proton indicates that the oxidative cyclization procedure applied leads exclusively to the formation of a single geometrical isomer, in this case the thermodynamically more stable *Z*-aurone [24]. In order to further support this hypothesis, *in silico* approaches were employed to perform energy calculations for selected aurones (**4a**, **4d**, **4h,** and **4i**). Results indicate that the *Z *isomers exhibit lower potential energy thus higher stability than their *E* counterparts. Differences in energy values between *E/Z* isomers are mainly attributed to torsional energy contribution (Table 1).

### 2.2. Biological Assays

All compounds were tested *in vitro* with human THP1-differentiated macrophages and cell viability evaluated by MTT assay (Table 1). The ability to inhibit the growth of the parasite without affecting macrophage viability is a good indication of selectivity of the compound, a desirable property for a drug candidate. The less cytotoxic aurones on the mammalian cells (IC_50_ > 20 *μ*M) were then assessed against the intracellular amastigote stage of *L. infantum*. The selectivity index (SI) was determined based on ratio of IC_50_ values measured on the mammalian cells and for the intracellular parasite (IC_50_ for mammalian cell line/IC_50_ for parasite). This ratio provides an indication of the selectivity of these compounds against *L. infantum* amastigotes when compared to normal cells. The results are shown in Table 2.

Aurones **4a**–**4f** bearing no substituents on ring A generally exhibited higher toxicity against THP1 cells than aurones **4g**–**4l **which possess oxygenated substituents on the same ring. Among the nonsubstituted on ring A compounds, aurone **4e** did not show significant toxicity but it is not a very active antileishmanial agent either (IC_50_ = 4.7 ± 0.5 *μ*M). The compounds possessing oxygenated substituents on positions 4 and 6 of ring A generally were less toxic against THP1 cells. However, aurone **4g**, one of the least toxic compound of the series, which possesses four methoxymethyl (MOM) groups, is a weak antiparasitic agent (IC_50_ = 5.1 ± 0.3 *μ*M).

The position and electronic nature of the substituent on ring B seem to significantly affect the activity of the compounds against *L. infantum* parasite as well as their cytotoxicity: the most active and one of the less toxic compounds of the series, 2′,4,6-trimethoxyaurone **4i** bears a methoxy group on position 2′ of ring B and exhibits potent antileishmanial activity with IC_50_ = 1.3 ± 0.1 *μ*M. Changing the electron-donating methoxy group to the nonoxygenated electron-donating methyl group on the same position results in compound **4j**, which exhibits comparable antileishmanial activity (IC_50_ = 1.6 ± 0.2 *μ*M), with **4i** but slightly higher toxicity. On the other hand, when position 2′ of ring B is occupied by an electron-withdrawing chlorine atom as in aurone **4k**, antiparasitic activity is substantially lower (IC_50_ = 12.2 ± 1.4 *μ*M) whereas the compound is not toxic to mammalian cells (in fact aurone **4k** is the least toxic compound of the series studied in this work). Aurone **4l **in which ring B is unsubstituted possesses significant antileishmanial activity (IC_50_ = 2.1 ± 0.9 *μ*M) although lower than the 2′-substituted analogues **4i **and **4j**.

Compounds **4i** and **4j** exhibited higher selectivity towards the intracellular form of the *L. infantum* parasite, when compared to the toxicity exerted on the mammalian cells (SI = 57.5  and 43.4, resp.). Amphotericin B was used as a reference antileishmanial drug currently applied on the chemotherapy of leishmaniasis. Its activity on the intracellular amastigotes was very similar with the one obtained with compounds **4i** and **4j**, although Amphotericin B was more toxic on the human-differentiated macrophages (SI = 20.6).

## 3. Experimental

^1^H NMR spectra and ^13^C NMR spectra were recorded on a Varian Gemini 2000 300 MHz spectrometer or a Varian 600 MHz spectrometer. Coupling constants (J) are expressed in hertz (Hz). Chemical shifts (*δ*) of NMR are reported in parts per million (ppm) units relative to the solvent. Melting points were determined on a Gallenkamp MFB-595 melting point apparatus and are uncorrected.

The LC/MS analysis was performed using Varian LC/MS using the ElectroSpray Ionisation technique and an Ion Trap detector.

### 3.1. General Procedure for the Synthesis of Chalcones 3j–3l

To a stirred solution of 2-hydroxy-4,6-dimethoxy-acetophenone (**1b**) (1eq) and a substituted benzaldehyde (1eq) in ethanol was added KOH (3eq, 20% w/v aqueous solution) and the mixture was stirred at room temperature for 24–72 h. The reaction mixture was cooled to 0°C (ice-water bath) and acidified with HCl (10% v/v aqueous solution). The precipitate formed was filtered and washed with 10% aqueous HCl solution.

**2′-Hydroxy-4′,6′-dimethoxy-2-methyl-chalcone (3j) [32]**

Prepared following the general procedure starting from 2-hydroxy-4,6-dimethoxy-acetophenone (**1b**, 500 mg, 2.3 mmol) and o-methylbenzaldehyde (278 mg, 2.3 mmol), dissolved in 9 mL ethanol and KOH (20% aqueous solution, 2 mL). The solid was recrystallized from methanol to afford yellow crystals. Yield: 420 mg (61%). mp 117–120°C; ^1^H NMR (CDCl_3_, 300 MHz)  *δ*: 14.26 (s, 1H), 8.08 (d, *J* = 15.3 Hz, 1H), 7.81 (d, *J* = 15.9 Hz, 1H), 7.64  (d, *J* = 6.6 Hz, 1H), 7.32–7.19 (m, 3H), 6.13 (s, 1H), 5.98 (s, 1H), 3.93  (s, 3H), 3.87  (s, 3H), 2.52  (s, 3H). ^13^C NMR (CDCl_3_, 75 MHz)  *δ* ppm 192.87, 168.54, 166.37, 162.65, 140.12, 138.33, 134.65, 131.01, 129.94, 128.73, 126.74, 126.40, 106.48, 93.90, 91.39, 55.99, 55.74, 20.12. MS (ESI) *m/z* [M+1]^+^ 299.

**2′-Hydroxy-4′,6′-dimethoxy-2-chloro-chalcone (3k)**

Prepared following the general procedure starting from 2-hydroxy-4,6-dimethoxy-acetophenone (**1b**, 500 mg, 2.3 mmol) and o-chlorobenzaldehyde (328 mg, 2.3 mmol), dissolved in 9 mL ethanol and KOH (20% aqueous solution, 2 mL). The solid was recrystallized from methanol to afford yellow crystals. Yield: 518 mg (70%). mp 135–136°C (lit. [33] mp 136–137°C); ^1^H NMR (CDCl_3_, 600 MHz)  *δ*: 14.19 (s, 1H), 8.13 (d, *J* = 15.6 Hz, 1H), 7.87 (d,  *J* = 15.6 Hz,  1H), 7.69–7.67 (m, 1H), 7.43–7.42 (m, 1H), 7.31–7.28 (m, 2H), 6.11 (d, *J* = 1.8 Hz, 1H), 5.95  (d, *J* = 2.4 Hz, 1H), 3.89  (s, 3H, OCH_3_), 3.83  (s, 3H, OCH_3_). ^13^C NMR (CDCl_3_, 75 MHz)  *δ*: 192.48, 168.62, 166.55, 162.62, 138.03, 135.53, 133.98, 130.83, 130.41, 130.18, 127.95, 127.12, 106.45, 93.94, 91.45, 56.03, 55.78. MS (ESI) *m/z* [M+1]^+^ 319.

**2′-Hydroxy-4′,6′-dimethoxy-chalcone (Flavokawain B, 3l)**

Prepared following the general procedure starting from 2-hydroxy-4,6-dimethoxy-acetophenone (**1b**, 500 mg, 2.3 mmol) and benzaldehyde (247 mg, 2.3 mmol), dissolved in 9 mL ethanol and KOH (20% aqueous solution, 2 mL). The solid was recrystallized from methanol to afford orange crystals. Yield: 460 mg (72%). mp 74–76°C (lit. [34] mp  89–90°C); ^1^H NMR (CDCl_3_, 600 MHz): *δ* 14.27 (s, 1H), 7.91 (d, *J* = 15.6 Hz, 1H), 7.79 (d, *J* = 15.6 Hz, 1H), 7.61 (dd, *J* = 7.8 Hz,   *J* = 1.2 Hz, 2H), 7.42–7.39 (m, 3H), 6.10 (d, *J* = 2.4 Hz, 1H, ), 5.96 (d, *J* = 2.4 Hz, 1H), 3.90 (s, 3H), 3.84 (s, 3H). ^13^C NMR (CDCl_3_, 75 MHz)  *δ*: 192.77, 168.54, 166.37, 162.63, 142.46, 135.68, 130.20, 129.01, 128.50, 127.65, 106.45, 93.90, 91.40, 55.99, 55.73. MS (ESI) *m/z *[M+1]^+^285.

### 3.2. General Procedure for the Preparation of *Z*-Aurones 4j–4k

To a solution of mercuric acetate (1.2eq) in pyridine was added chalcone (1eq) at room temperature and the mixture was stirred at 110°C for 1 h. The cooled reaction mixture was poured into ice cold water and acidified with HCl (10% aqueous solution). The precipitated solid was extracted with dichloromethane, the extracts were dried (Na_2_SO_4_) and the solvent was evaporated to give a solid which was further purified by recrystallization from methanol/hexane.

**4, 6-Dimethoxy-2′-methylaurone (4j)**

Prepared following the general procedure starting from chalcone **3j**  (200 mg, 0.67 mmol) in 7 mL pyridine. After recrystallization, the product was obtained as yellow crystals. Yield: 112 mg (48%). mp 180–183°C (lit. [21] mp 194–195°C); ^1^H NMR  (CDCl_3_, 300 MHz)  *δ*: 8.15  (d, *J* = 6 H*z*, 1H), 7.32–7.23 (m, 4H,), 7.00 (s, 1H), 6.39 (d, *J* = 1.8 Hz, 1H), 6.15 (d,  *J* = 1.8 Hz,  1 H), 3.98 (s, 3H), 3.93 (s, 3H), 2.52 (s, 3H). ^13^C NMR (CDCl_3_, 75 MHz)*δ*: 180.86, 169.30, 169.09, 159.57, 148.14, 138.96, 131.21, 130.90, 130.74, 129.42, 126.35, 124.73, 107.95, 105.41, 94.17, 89.33, 56.37, 56.28, 20.48.

**4, 6-Dimethoxy-2′-chloro-aurone (4k)**

Prepared following the general procedure starting from chalcone **3k** (250 mg, 0.78 mmol) in 7.3 mL pyridine. After recrystallization, the product was obtained as yellow crystals. Yield: 194 mg (78%). mp 191–193°C (lit. [27] mp 201–203°C); ^1^H NMR (CDCl_3_, 300 MHz) *δ*: 8.26 (dd, *J* = 7.5 Hz, *J* = 1.8 Hz, 1 H), 7.45 (dd, *J* = 7.8 Hz, *J* = 1.5 Hz, 1H), 7.38–7.28 (m, 2H), 7.22 (s, 1H), 6.39 (d, *J* = 1.8 Hz, 1H), 6.16 (d, *J* = 1.8 Hz, 1H), 3.98 (s, 3H), 3.93 (s, 3H). ^13^C NMR (CDCl_3_, 75 MHz) *δ*: 180.47, 169.28, 169.22, 159.74, 148.90, 135.70, 132.02, 130.87, 130.19, 130.08, 127.02, 106.07, 105.26, 94.33, 89.50, 56.41, 56.29. MS (ESI) m/z [M+1]^+^ 317.

**4, 6-Dimethoxyaurone (4l)**

Prepared following the general procedure starting from chalcone **3l** (200 mg, 0.70 mmol) in 6.6mL pyridine. After recrystallization, the product was obtained as yellow crystals. Yield: 88 mg (44%). mp 139–142°C (lit. [34] mp 152–153°C); ^1^H NMR (CDCl_3_, 300 MHz) *δ*: 7.87 (d, *J* = 7.6 Hz, 2 H), 7.46–7.36 (m, 3 H), 6.78 (s, 1H), 6.40 (d, *J* = 1.8 Hz, 1 H), 6.15 (d, *J* = 1.8 Hz, 1 H), 3.98 (s, 3 H), 3.94 (s, 3H); ^13^C NMR (CDCl_3_, 75 MHz) *δ*: 180.85, 169.25, 169.16, 159.60, 148.03, 132.74, 131.25, 131.15, 129.46, 128.92, 110.93, 105.40, 94.22, 89.41, 56.38, 56.26. MS (ESI) *m/z* [M+1]^+^ 283.

### 3.3. Parasite and Cell Cultures

#### 3.3.1. Parasites

A cloned line of *L. infantum* (MOM/MA671 TMAP263) promastigotes, stably expressing the luciferase gene (LUC), was grown in RPMI 1640 medium (Bio Whitaker, Belgium) supplemented with 10% heat-inactivated fetal bovine serum (FBS) (Bio Whitaker, Belgium), 2 mML-glutamine (Bio Whitaker, Belgium), 20 mM Hepes (Bio Whitaker, Belgium), 100 U/mL penicillin, and 100 *μ*g/mL streptomycin (Bio Whitaker, Belgium). Selection of LUC positive parasites was done by adding geneticin-sulphate (Sigma-Aldrich, USA) to the culture media at a final concentration of 5 *μ*g/mL. Parasites were maintained in culture, at 26°C by subpassage (10°C parasites/mL) at every 5 days.

*L. infantum* axenic amastigotes, stably expressing the LUC gene were derived from promastigotes by culturing them in MAA (Medium for Axenic Amastigotes) culture medium. MAA consisted of a modified medium 199 with Hanks balanced salt solution (Gibco-Invitrogen, Spain) supplemented with 0.5% soya broth trypto-casein (Bio-Rad, UK), 15 mM D-glucose (Panreac, Spain), 4 mM NaHCO_3_ (Sigma-Aldrich, USA). The pH was adjusted to 5.8, and the media was 0.2 *μ*m sterilized by filtration and further supplemented with 0.023 mM bovine hemin (Fluka, USA), 5 mM L-glutamine, and 25% of heat inactivated FBS. Amastigotes were maintained in culture, at 37°C in an atmosphere containing 5% CO_2_ by subpassage (10^5^  parasites/mL), at every 5 days.

#### 3.3.2. Cell Lines

The human leukaemia monocyte THP1 cell line was cultured as a monolayer at 37°C in a humidified atmosphere containing 5% CO_2_. Cells were grown in RPMI 1640 medium, supplemented with 10% heat-inactivated FBS, 2 mM L-glutamine, 100 U/mL penicillin, and 100 *μ*g/mL streptomycin, and were maintained in culture, by subpassage every 3 days. 

### 3.4. Toxicity of Aurones on THP1-Differentiated Macrophages

For macrophage differentiation, human leukaemia monocyte cell line (THP-1 cells) cells were incubated in the presence of 20 ng/ml phorbol 12-myristate 13-acetate (PMA, Sigma-Aldrich) for 18 h at 37°C, 5% CO_2, _and left another 24 h with fresh medium containing no PMA to induce maturation. Serial dilutions of each aurone, ranging from 100 to 1.56 *μ*M in culture media, were added to the wells, in quadruplicate, and incubated for 72 h at 37°C, 5% CO_2_. Cell viability was assessed by the MTT assay, as described before [35].

The IC_50_ value, that is, the concentration of the aurone necessary to decrease cell viability to 50% of the untreated control, was determined by linear regression analysis.

### 3.5. Growth Inhibition Assays

THP1-differentiated macrophages were infected for a period of 4 h with stationary phase LUC expressing, *L. infantum* axenic amastigotes, at a 5 : 1 parasite to cell ratio. After the infection period, cells were washed with culture media to remove noninternalized parasites. Serial dilutions of the aurones, ranging from 50 to 0.78 *μ*M in culture media, were added to the wells, in quadruplicate, and incubated for 72 h at 37°C, 5% CO_2_. After the incubation period, the luciferase activity of intracellular amastigotes was determined as described before [36]. The percentage of growth inhibition was calculated as described above.

### 3.6. Molecular Modeling Calculations

Molecular Modeling calculations were performed using MacroModel module of Schrodinger package, (Schrodinger, LLC, New York, NY, 2011). *E* and *Z *isomers for the four selected aurones were initially energetically minimized using Molecular Mechanics with OPLS_2005 force field and CHCl_3_ as solvent, simulating the environment of the NMR solvent. Minimization was performed with PRCG “Polak-Ribiere Conjugate Gradient” algorithm using 1000 iterations and an energy tolerance of 0.05 kcal/mol^−1^ Å^−1^, to reach a local minimum.

## 4. Conclusions

A series of substituted aurones have been synthesized and tested for their antileishmanial activity against the intracellular form of *L. infantum* parasite as well as their cytotoxicity against mammalian cells. The position of the substituents as well as their electronic nature seem to play an important role in the antiparasitic activity. Among the twelve tested compounds, the aurones **4i **and **4j**, possessing methoxy groups at positions 4 and 6 of ring A and electron-donating groups on position 2′of ring B exhibited antiparasitic activity comparable to that of Amphotericin B. The significant antileishmanial activity of aurones **4i **and **4j** is combined with low cytotoxicity, thus rendering these compounds eligible for further studies toward the design and synthesis of novel antileishmanial agents possessing this naturally occurring “privileged” scaffold.

## Figures and Tables

**Table 1:** Energy calculation of selected energetically minimized aurones (kJ mol^−1^).

Compound	Potential energy	Torsional energy
**4a** (*Z*-isomer)	88.08	−1.82
**4a** (*E*-isomer)	110.16	12.14
**4d** (*Z*-isomer)	107.57	13.54
**4d** (*E*-isomer)	117.81	13.18
**4h** (*Z*-isomer)	139.95	22.83
**4h** (*E*-isomer)	162.43	37.43
**4i** (*Z*-isomer)	132.81	0.76
**4i** (*E*-isomer)	169.79	38.41

**Table 2:** Antileishmanial activity and cytotoxicity of the synthesized aurones.

Compound	IC_50 _(*μ*M)		Selectivity index (SI)^a^
	*L. infantum* intracellular amastigotes	THP1-differentiated macrophages
**4a**	nd^b^	16.5 ± 3.1	nd
**4b**	nd	8.5 ± 0.8	nd
**4c**	nd	14.5 ± 2.1	nd
**4d**	nd	17.5 ± 3.1	nd
**4e**	4.7 ± 0.5	54.2 ± 7.3	11.5
**4f**	nd	20.5 ± 1.2	nd
**4g**	5.1 ± 0.3	62.5 ± 1.3	12.4
**4h**	nd	19.6 ± 2.5	nd
**4i**	1.3 ± 0.1	75.4 ± 4.7	57.5
**4j**	1.6 ± 0.2	68.1 ± 2.1	43.4
**4k**	12.2 ± 1.4	>100	>8.2
**4l**	2.1 ± 0.9	57.5 ± 3.4	26.9
Amphotericin B	1.2 ± 0.1	23.8 ± 2.3	20.6

^a^The selectivity index represents the ratio of IC_50_ on THP1-differentiated macrophages to the IC_50_ on intracellular parasite.

^b^nd: not determined, for compounds with IC_50 _values below 20 *μ*M on THP1-differentiated macrophages.
